# Metabolites from Marine Macroorganisms of the Red Sea Acting as Promoters or Inhibitors of Amylin Aggregation

**DOI:** 10.3390/biom14080951

**Published:** 2024-08-06

**Authors:** Mawadda Alghrably, Mohamed A. Tammam, Aikaterini Koutsaviti, Vassilios Roussis, Xabier Lopez, Giulia Bennici, Abeer Sharfalddin, Hanan Almahasheer, Carlos M. Duarte, Abdul-Hamid Emwas, Efstathia Ioannou, Mariusz Jaremko

**Affiliations:** 1 Division of Biological and Environmental Sciences and Engineering (BESE), King Abdullah University of Science and Technology (KAUST), Thuwal 23955-6900, Saudi Arabia; mawadda.alghrably@kaust.edu.sa (M.A.); giulia.bennici@kaust.edu.sa (G.B.); 2 Section of Pharmacognosy and Chemistry of Natural Products, Department of Pharmacy, School of Health Sciences, National and Kapodistrian University of Athens, 15771 Athens, Greece; mtammam@pharm.uoa.gr (M.A.T.); kkoutsaviti@pharm.uoa.gr (A.K.); roussis@pharm.uoa.gr (V.R.); 3 Department of Biochemistry, Faculty of Agriculture, Fayoum University, Fayoum 63514, Egypt; 4 Polimero eta Material Aurreratuak: Fisika, Kimika eta Teknologia, Kimika Fakultatea, UPV/EHU & Donostia International Physics Center (DIPC), PK 1072, 20018 Donostia-San Sebastian, Euskadi, Spain; xabier.lopez@ehu.es; 5 Department of Chemistry, Faculty of Science, King Abdulaziz University, P.O. Box 80203, Jeddah 21589, Saudi Arabia; sharfalddin.aa@hotmail.com; 6 Department of Biology, College of Science, Imam Abdulrahman Bin Faisal University (IAU), Dammam 31441-1982, Saudi Arabia; halmahasheer@iau.edu.sa; 7 Red Sea Research Center, Division of Biological and Environmental Sciences and Engineering, King Abdullah University of Science and Technology, Thuwal 23955-6900, Saudi Arabia; carlos.duarte@kaust.edu.sa; 8 Core Lab of NMR, King Abdullah University of Science and Technology (KAUST), Thuwal 23955-6900, Saudi Arabia; abdelhamid.emwas@kaust.edu.sa

**Keywords:** marine natural products, Red Sea, amylin aggregation

## Abstract

Amylin is part of the endocrine pancreatic system that contributes to glycemic control, regulating blood glucose levels. However, human amylin has a high tendency to aggregate, forming isolated amylin deposits that are observed in patients with type 2 diabetes mellitus. In search of new inhibitors of amylin aggregation, we undertook the chemical analyses of five marine macroorganisms encountered in high populations in the Red Sea and selected a panel of 10 metabolites belonging to different chemical classes to evaluate their ability to inhibit the formation of amyloid deposits in the human amylin peptide. The thioflavin T assay was used to examine the kinetics of amyloid aggregation, and atomic force microscopy was employed to conduct a thorough morphological examination of the formed fibrils. The potential ability of these compounds to interact with the backbone of peptides and compete with β-sheet formation was analyzed by quantum calculations, and the interactions with the amylin peptide were computationally examined using molecular docking. Despite their structural similarity, it could be observed that the hydrophobic and hydrogen bond interactions of pyrrolidinones **9** and **10** with the protein sheets result in one case in a stable aggregation, while in the other, they cause distortion from aggregation.

## 1. Introduction

Extreme conditions in ocean habitats have driven marine organisms to biosynthesize a wide range of unique and valuable bioactive compounds, enabling adaptation to hypoxia, low and high temperatures, and high salinity [1] to satisfy their ecological needs [2]. Bioprospecting is targeting the discovery of bioactive natural products to be utilized in drug development, as nutraceuticals and as cosmeceuticals, as well as in other industrial applications. Up to now, approximately 20 medications currently approved for use in patients are produced using naturally occurring compounds originating from marine sources [3]. Among these, 12 are used to treat various forms of human cancer [3,4]. Furthermore, many marine natural products exhibit a broad spectrum of bioactivities, supporting applications ranging from treating allergies to tackling diabetes, while others display high potential for use as functional food ingredients [5,6,7,8,9].

The Red Sea is characterized by its extreme conditions, including exceptionally high temperatures (up to 31 °C in the summer on the surface and 22 °C minimum temperature at 3 km of depth), high salinity (ca. 42‰ in the northern Red Sea), and low oxygen concentration [10,11]. Even though the Red Sea harbors a large number of corals, sponges, seaweeds, and jellyfish, the chemodiversity of these marine organisms remains relatively underexplored compared to that of other coral reef oceans and marine habitats worldwide [12,13]. According to the literature, less than 1000 marine natural products have been isolated from Red Sea organisms [14]. The search for bioactive marine natural products remains exploratory, aiming to identify extracts with interesting chemical profiles, which are then subjected to a series of tests to assess their bioactivity against specific targets.

Amylin, also known as human islets amyloid polypeptide, is a 37-amino acid residue peptide stored inside the β-cells of the pancreas and is part of the endocrine pancreatic system that contributes to glycemic control. The peptide is co-stored and co-secreted with insulin into the blood circulation. The main role of amylin is to regulate blood glucose levels by lowering gastric emptying and promoting satiety, thereby preventing post-prandial spikes in blood glucose levels. However, it is well known that human amylin has a high tendency to aggregate and form isolated amylin deposits, which are observed mainly in the islets of Langerhans of patients with type two diabetes mellitus. Insulin resistance is associated with the overexpression of islet amyloid polypeptide (IAPP), which can aggregate both intra- and extracellularly into giant insoluble amyloid fibrils and small soluble amyloid aggregates. These amylin aggregates have been implicated in the disruption of the cellular membrane of β-cells by generating stress related to the membrane as a result of the unregulated influx of ions into the cells. Some preclinical research studies showed that amylin might bind selectively to the Postrema region, a specific brain region. As a result, it is regarded as a neuroendocrine hormone that plays a crucial part in controlling the pace at which glucose enters the bloodstream. Numerous biochemical studies have revealed that the region ^22^NFGAILS^28^ of the amino acid sequence of hIAPP is responsible for the protein’s aggregation tendencies. This region has a substantial contribution to the process of amyloid formation [15,16,17]. Hence, the search for natural products that promote or inhibit amylin aggregation is of interest to drug development.

**Figure 1:**
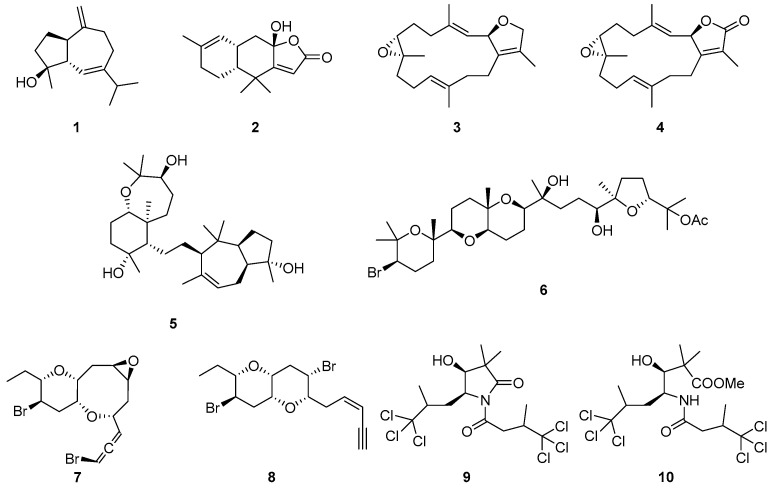
Metabolites **1**–**10** isolated from marine macroorganisms of the Red Sea and evaluated in this study.

Herein, we isolated 10 natural products belonging to different chemical classes from five marine macroorganisms frequently encountered in the Red Sea (**1**–**10**, Figure 1) and proceeded to evaluate their activity on amylin aggregation, aiming to shed light on the potential factors underpinning the varying activities observed.

## 2. Materials and Methods

### 2.1. Materials

C-Protected disulfide-bridged human amylin (KCNTATCATQRLANFLVHSSNNFGAILSSTNVGSNTY-NH_2_) was purchased from Kare BayBiochem (certified purity of 99.30%) and used as received.

### 2.2. Extraction and Isolation of Metabolites

Specimens of the soft coral *Lobophytum* sp. (ATPH/MP0641) were collected by SCUBA diving from Al Fahal reefs off Thuwal, Saudi Arabia, at a depth of 10–12 m in January 2018. The fresh specimens were exhaustively extracted with mixtures of CH_2_Cl_2_/MeOH at room temperature. After evaporation of the solvent in vacuo, the organic extract (1.8 g) was subjected to vacuum column chromatography over silica gel using cHex with increasing amounts of EtOAc and finally MeOH as mobile phase to yield 11 fractions (A–K). Fraction C (160.8 mg) was subjected to gravity column chromatography over silica gel using mixtures of cHex and EtOAc of increasing polarity as eluent to afford 11 fractions (C1–C11), among which C6 was identified as **1** (22.5 mg).

Specimens of the soft coral *Sarcophyton* sp. (ATPH/MP0639) were collected by SCUBA diving from Al Fahal reefs off Thuwal, Saudi Arabia, at a depth of 6–8 m in January 2018. The fresh specimens were exhaustively extracted with mixtures of CH_2_Cl_2_/MeOH at room temperature. After evaporation of the solvent in vacuo, the organic extract (9.5 g) was subjected to vacuum column chromatography over silica gel using cHex with increasing amounts of EtOAc and finally MeOH as mobile phase to yield 12 fractions (A–L), among which F and G were identified as **3** (996.7 mg) and **4** (372.0 mg).

Specimens of the sponge *Siphonochalina siphonella* (ATPH/MP0669) were collected by SCUBA diving from Rose Reef off the village of Thuwal in Saudi Arabia, at a depth of 10–12 m in January 2018. The fresh specimens were exhaustively extracted with mixtures of CH_2_Cl_2_/MeOH at room temperature. After evaporation of the solvent in vacuo, the organic extract (0.4 g) was subjected to gravity column chromatography over silica gel using cHex with increasing amounts of EtOAc and finally MeOH as mobile phase to yield 15 fractions (A–O). Fractions K and L were pooled together (91.2 mg) and subjected to gravity column chromatography over silica gel using cHex/Μe_2_CO (82:18) as eluent to obtain 5 fractions (K1-K5). Fraction K2 (48.6 mg) was further purified by normal-phase HPLC using cHex/EtOAc (75:25) as eluent to yield **5** (10.9 mg).

Specimens of the sponge *Lamellodysidea* sp. (ATPH/MP0649) were hand-picked off the village of Thuwal in Saudi Arabia, at a depth of 1–2 m in January 2018. The fresh specimens were extensively extracted with mixtures of CH_2_Cl_2_/MeOH at room temperature. After evaporation of the solvent in vacuo, the organic extract (15.8 g) was subjected to vacuum column chromatography on silica gel, using cHex with increasing amounts of EtOAc, followed by EtOAc with growing amounts of MeOH as mobile phase, to yield seven fractions (A–G). Fractions B and C were combined (4.95 g) and fractionated by gravity column chromatography on silica gel, using a mixture of cHex and EtOAc of increasing polarity as mobile phase to afford 16 fractions (B1–B16). Fraction B9 (3.4 g) was fractionated by gravity column chromatography on silica gel using mixtures of toluene and Me_2_CO of increasing polarity as mobile phase to yield fourteen fractions (B9a-B9n). Fraction B9g (634.6 mg) was subjected repetitively to normal-phase HPLC, using toluene/Me_2_CO (96:4) and cHex/Me_2_CO (90:10) as eluent to afford **9** (319.3 mg) and **10** (22.4 mg). Fraction B9k (192.7 mg) was subjected to normal-phase HPLC, using cHex/EtOAc (80:20) as eluent, to yield **2** (9.2 mg).

Specimens of the red alga *Laurencia* sp. (ATPH/MP0677) were collected by hand from Rose Reef off the village of Thuwal in Saudi Arabia, at a depth of 1.5–2 m in January 2018. The fresh specimens were exhaustively extracted with mixtures of CH_2_Cl_2_/MeOH at room temperature. After evaporation of the solvent in vacuo, the organic extract (288.0 mg) was subjected to vacuum column chromatography over silica gel using cHex with increasing amounts of EtOAc and finally MeOH as mobile phase to yield 7 fractions (A–G), among which **6** (8.0 mg) was isolated in pure form. Fraction A (102.8 mg) was subjected to vacuum liquid chromatography over silica gel using mixtures of *n*Hex and EtOAc of increasing polarity as eluent to afford 7 fractions (A1–A7). Fraction A2 (37.9 mg) was repeatedly purified by normal-phase HPLC using cHex/EtOAc (98:2) as mobile phase to afford **8** (1.5 mg). Fraction B (65.6 mg) was subjected to normal-phase HPLC using cHex/EtOAc (83:17) and subsequently cHex/Me_2_CO (95:15) as mobile phase to afford **7** (33.0 mg).

Metabolites **1**–**10** were identified as alismol (**1**) [18], furodysinin lactone (**2**) [19], (2*R*,7*R*,8*R*)-sarcophytoxide (**3**) [20], sarcophine (**4**) [21], sipholenol A (**5**) [22], 23-acetylthyrsiferol (**6**) [23], thuwalallene A (**7**) [23], thuwalenyne A (**8**) [23], dysidamide (**9**) [24] and 7,7,7-trichloro-3-hydroxy-2,2,6-trimethyl-4-(4,4,4-trichloro-3-methyl-1-oxobutylamino)-heptanoic acid methyl ester (**10**) [25] by comparison of their spectroscopic and physical characteristics with those reported in the literature.

### 2.3. Thioflavin T (ThT) Assay

The studies of the amyloid aggregation kinetics of human amylin in the presence of the tested compounds were conducted in a 96-well flat-bottom black plate (Corning 3915) sealed with transparent film (Duck Brand Crystal Clear Tape, Avon, OH, USA). All experiments were carried out at a temperature of 25 °C with samples containing 20 μM thioflavin T (ThT), 2% (*v*/*v*) DMSO (final concentration), and a 50 mM HEPES buffer solution (pH 7.4), using a final human amylin concentration of 40 μM (from a 10 mg/mL stock in 100% DMSO), and a final concentration of 40 μM for each compound, in a final volume of 200 μL. These conditions are widely accepted for studying the aggregation properties of amylin [26], and, therefore, we applied an analogous procedure to study amylin with natural compounds. The aggregation assay was performed on a Synergy 2 Multi-Mode Microplate with excitation set at 485/20 nm and emission at 528/20 nm with a gain equal to 35. The measurements were collected at 50% top reading, each 5 min after a 3 s vibration.

### 2.4. Atomic Force Microscopy (AFM)

AFM was conducted in a 256-scanning line at a 1 Hz scanning rate, using Bruker’s Nano Dimension Icon (Bruker Nano GmbH, Berlin, Germany). In this procedure, 40 μM hIAPP samples were incubated with and without the tested compounds in a 1:1 ratio at 25 °C for 24 h. All the samples were dissolved in 50 mM HEPES buffer. The heights of peptide aggregates were obtained from the section tool of NanoScope Analysis 1.5, and the line positions with average effects in the images were selected, except for peptides alone in fibril form.

### 2.5. Quantum Calculations

To study the interaction energies of the different complexes formed by the peptides and compounds **9** and **10**, quantum mechanical calculations were performed using the Gaussian 16 program [27]. All calculations were made at the Density Functional Theory (DFT) level, using B3LYP functional [28,29] and Pople’s 6-31+G(d,p) basis set [30], and dispersion interactions were considered with the empirical D3 version of Grimme’s dispersion with Becke–Johnson damping [31]. All geometry optimizations were carried out in the solvent phase, using the Polarizable Continuum Model approach [32] to take solvation effects into account.

### 2.6. Molecular Docking Platforms

The docking process was performed using Molecular Operating Environment (MOE) software (2019). The investigation was done as in the serine protease inhibitor study [33]. The crystal structure of hIAPP obtained by an NMR study at physiological pH in a membrane environment (PDB ID:2L86) was downloaded from the database RCSB PDB [34]. Initially, the protein was prepared using the MOE-Quick prep feature to optimize, minimize the energy via 0.30 °A RMSD, and correct the missing H or distribution of partial charges [35,36]. Moreover, the structures of the investigated compounds and epigallocatechin gallate (EGCG) were optimized and saved as a database. Afterward, the docking process was started under the default option, where the compound was placed using the triangle matcher method, and GBVI/WSA dG and London dG were used for rescoring and scoring, respectively. The extracted results were shown with a corresponding binding energy measured in units of kcal/mol.

## 3. Results

In the context of the present investigation, two sesquiterpenes (**1** and **2**), two diterpenes (**3** and **4**), two triterpenes (**5** and **6**), two C_15_ acetogenins (**7** and **8**), and two pyrrolidinones (**9** and **10**) were isolated from five marine macroorganisms of the Red Sea and were selected for evaluation of their activity on amylin aggregation. Specifically, compound **1** was isolated from a soft coral of the genus *Lobophytum*, compounds **3** and **4** were isolated from a soft coral of the genus *Sarcophyton*, compound **5** was isolated from the sponge *Siphonochalina siphonella*, compounds **2**, **9**, and **10** were isolated from a sponge of the genus *Lamellodysidea* and compounds **6**–**8** were isolated from a red alga of the genus *Laurencia*.

**Figure 2:**
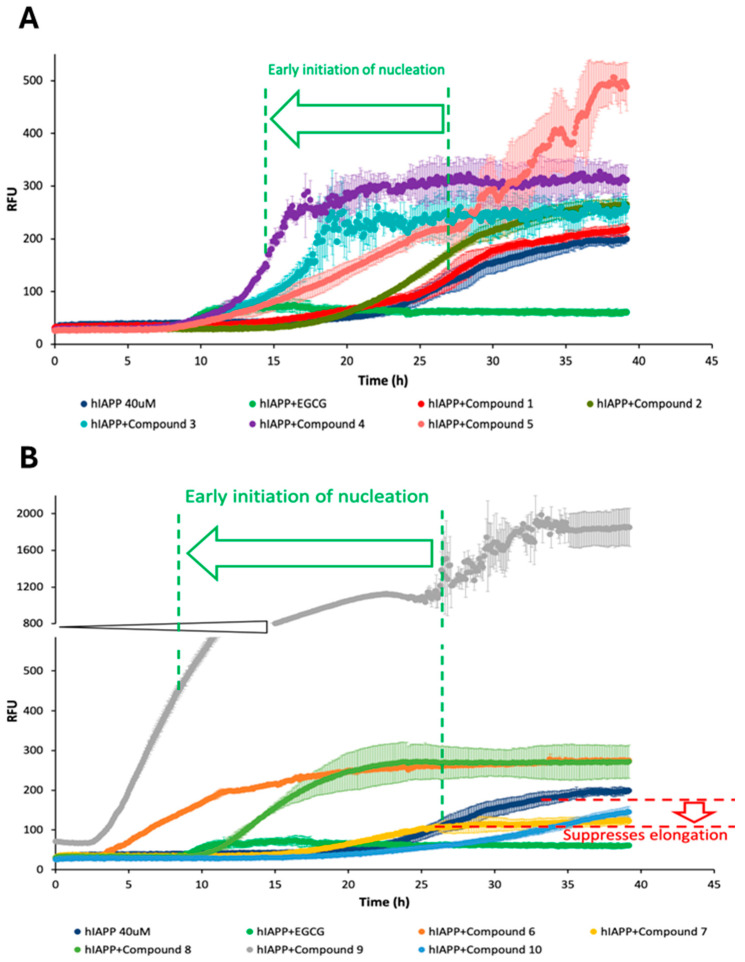
The effect of (**A**) compounds **1**–**5** and (**B**) compounds **6**–**10** on hIAPP aggregation in HEPES buffered solution for 40 h at 25 °C. hIAPP (dark blue) and EGCG (green) were used as a reference point for the compounds.

The aggregation of human amylin was evaluated in the presence of each compound to examine the effects of the different natural compounds on the kinetics and the structure of the peptide (Figure 2).

Based on the data obtained from the ThT assay, it was observed that the minimal effect on the aggregation of hIAPP resulted in interaction with compound **1**. The fluorescence intensity for compound **1** is almost the same as that for the peptide alone. Compounds **7** and **10** led to a suppression of hIAPP aggregation and may be considered aggregation inhibitors, with an effect weaker than that of EGCG used as a reference. On the other hand, compounds **2**–**6**, **8**, and especially **9** may be considered aggregation promoters since a significant increase in fluorescence intensity is observed upon their addition to the peptide.

**Figure 3:**
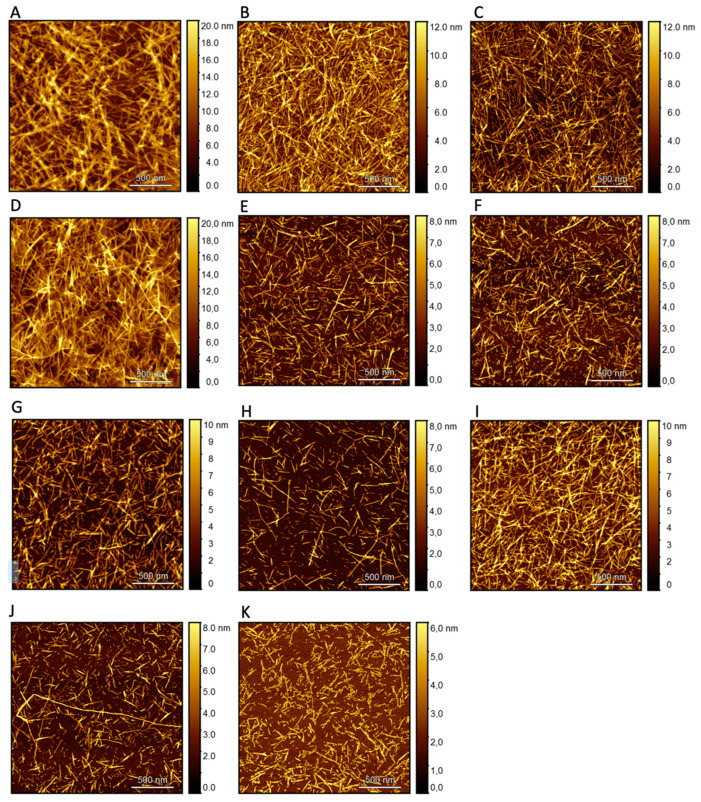
Solid-state AFM images of hIAPP with the tested compounds in a 1:1 ratio. Samples dissolved in HEPES pH 7.4 and incubated for 24 h at 25 °C. A 2 × 2 µm image of the formed fibrils for (**A**) Positive control and point of reference hIAPP 40 µM, (**B**) hIAPP and compound **1**, (**C**) hIAPP and compound **2**, (**D**) hIAPP and compound **3**, (**E**) hIAPP and compound **4**, (**F**) hIAPP and compound **5**, (**G**) hIAPP and compound **6**, (**H**) hIAPP and compound **7**, (**I**) hIAPP and compound **8**, (**J**) hIAPP and compound **9**, (**K**) hIAPP and compound **10**.

Atomic Force Microscopy (AFM) analysis (Figure 3) supported the results of the ThT. The sample composed of hIAPP and compound **9** (Figure 3J) exhibits the most extensive and dense fibrils among all the samples, in line with the ThT results, which show the highest fluorescence intensity and indicate the promotion of aggregation in the presence of compound **9**. On the other hand, the sample composed of hIAPP and compound **10** (Figure 3K) has the shortest fibrillar structure, a result that confirms the weakening of the fibril elongation process in comparison to the control and the other samples.

**Figure 4:**
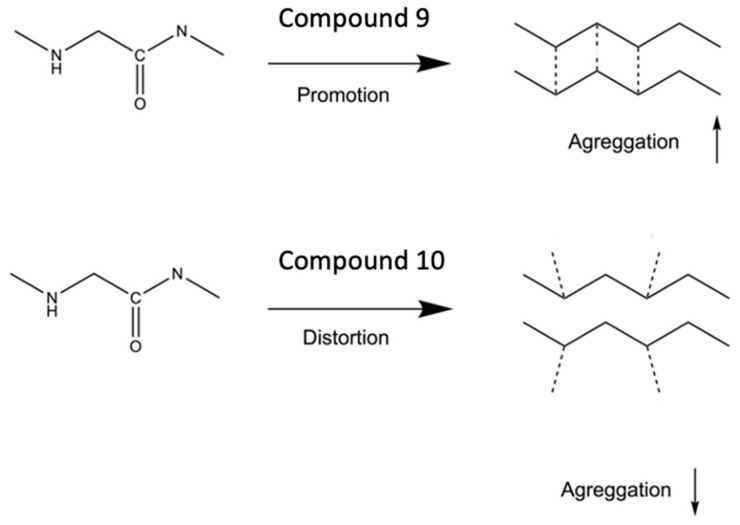
Schematic illustration of the promotion and distortion of the aggregation process by compounds **9** and **10**.

Compound **9** is a pyrrolidinone with a hydrogen bond donor (–OH) and a hydrogen bond acceptor (O). Therefore, we hypothesize that compound **9** can easily be integrated between the β-sheets of the peptide, thus facilitating the formation of hydrogen bonds acting as an aggregation promoter. On the other hand, the chemical structure of compound **10** lacks the rigid, stabilized 5-membered ring with the hydrogen bond acceptor. Instead, this pyrrolidinone has a more linear structure than its counterpart **9**, which contains the cyclic moiety. As such, compound **10** has more conformational freedom, making it difficult to form a stable hydrogen bonds network with the β-sheets of the peptide (Figure 4). Thus, the potential acceptor (O) in the carboxyl group is too flexible. This flexibility reduces the possibility of hydrogen formation in contrast to compound **9**, where the carbonyl oxygen can efficiently be used as an acceptor for hydrogen bonds. Furthermore, the hydrogen bond donor (–OH) in compound **10** competes with other peptide molecules for the β-sheet formation and most likely integrates between them, making the β-sheet fibril formation more difficult. Also, the absence of the ring in compound **10** gives it a more flexible rotation, and the presence of multiple chloride atoms turns the compound into a great acceptor for potential hydrogen bonds in between β-sheets.

The affinity of the tested compounds to bind the hIAPP is presented in Table 1. More negative values for docking scores indicate stronger bond interactions. Interestingly, compounds **9**, **10**, and **6** showed good scores (−5.91, −6.156 and −7.397 kcal/mol, respectively). Among them, triterpene **6** has the best binding energy, even better than the used reference (EGCG).

## 4. Discussion

Concerning the ThT assay, the tests were carried out in HEPES buffer at pH 7.4, monitored by ThT fluorescence for 40 h, with the use of hIAPP as a positive control and EGCG as a negative control, reducing the β-sheet content of all protein aggregates and preventing the formation of amyloidogenic fibrils and aggregates [37,38]. The results revealed that compound **1** had minimal effect on the aggregation of hIAPP, as its fluorescence intensity closely resembled that of the peptide alone used as a positive control (Figure 2). Upon interaction with compounds **5** and **9**, the fluorescence intensity of hIAPP was significantly higher, showing a steep increase in the elongation phase (Figure 2). Compounds **7** and **10** were identified as aggregation inhibitors, although their effect was weaker than that of EGCG. Conversely, compounds **2**–**6**, **8**, and **9** were considered aggregation promoters, as their fluorescence intensity exhibited a significant increase upon their addition to the peptide, with compound **9** showing particularly pronounced effects. The amyloid formation included three phases: nucleation phase, elongation phase, and saturation phase. Upon interaction with each compound, the representation of the nucleation and elongation phases of hIAPP aggregation differed. These phases are reported in Table 2, where the nucleation and the elongation phases are categorized according to the strength of the effect observed.

The findings from the ThT assay were supported by the AFM analysis. AFM was used to analyze the topography of the solution samples of hIAPP with each of the isolated compounds on mica surfaces after 24 h of incubation. This experiment confirmed the presence of differently distributed amyloid fibrils consisting of hIAPP-compound complexes (Figure 3). In the case of the hIAPP solution, which was used as a positive control, the fibril aggregates were clearly observable at high intensity using AFM (Figure 3A).

All samples incorporating different compounds exhibited aggregation and developed fibrils in a size and shape remarkably similar to the control, except for compounds **9** and **10**, which showed fibrils with a unique morphology (Figure 3J,K). The sample composed of hIAPP and compound **9** (Figure 3J) exhibited the most extensive and dense fibrils among all samples, in line with the ThT results where the highest fluorescence intensity was observed, indicating the promotion of aggregation in the presence of compound **9**. However, it is evident that the packing of fibrils in this case is lower than that in the control sample. On the other hand, the sample composed of hIAPP and compound **10** (Figure 3K) had the shortest fibrillar structure, a result that confirmed the weakening of the fibril elongation process in comparison to the control and the other samples (Figure 2).

**Figure 5:**
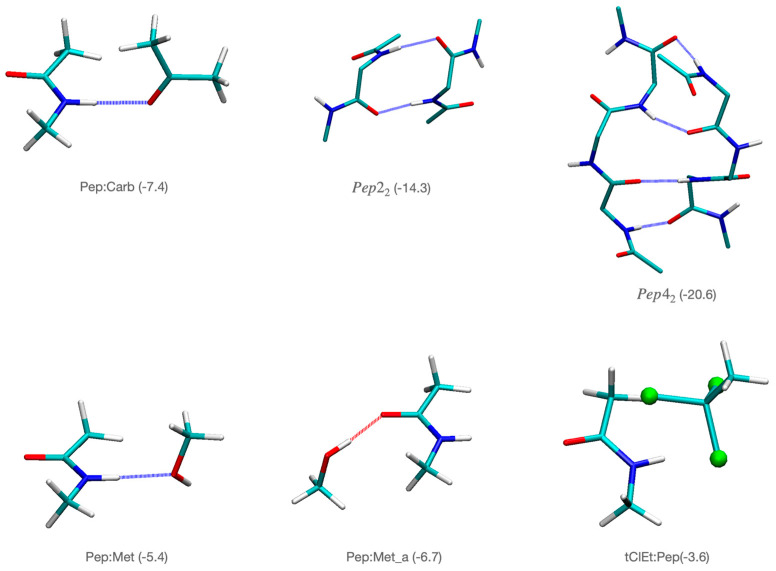
Interaction energies in kcal/mol calculated at the B3LYP-B3DJ(PCM)/6-31+G(d,p) to estimate the stabilization introduced by the presence of hydrogen bonds in ß-sheet structures and between different functional groups present in compounds **9** and **10** with the peptide bond. Pep_n_ stands for a chain with n peptide bonds, Met for methanol, and tClEt for ethane trichloride. Notice that both compounds **9** and **10** show the presence of these functional groups (alcohol, carbonyl within an amide bond, and trichloride aliphatic groups) in their structures. Interaction energies were calculated as the difference in energy between the dimer and the corresponding monomers (see methods for details).

**Figure 6:**
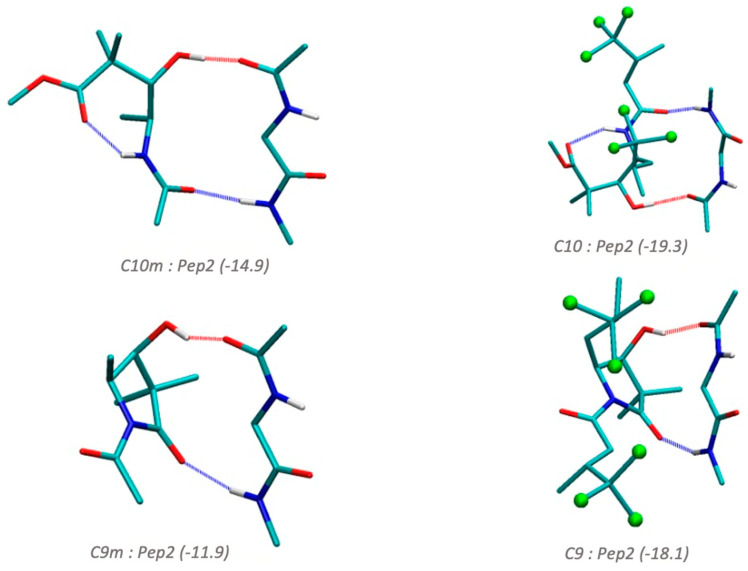
Interaction energies in kcal/mol calculated at the B3LYP-B3DJ(PCM)/6-31+G(d,p) for compounds **9** and **10** with different peptide models. Pep2 stands for a glycine dipeptide in a ß-sheet conformation. C9m and C10m are derivatives of compounds **9** and **10** in which the trichloride chains are omitted. Hydrogens that do not participate in hydrogen bonds are omitted for clarity.

**Figure 7:**
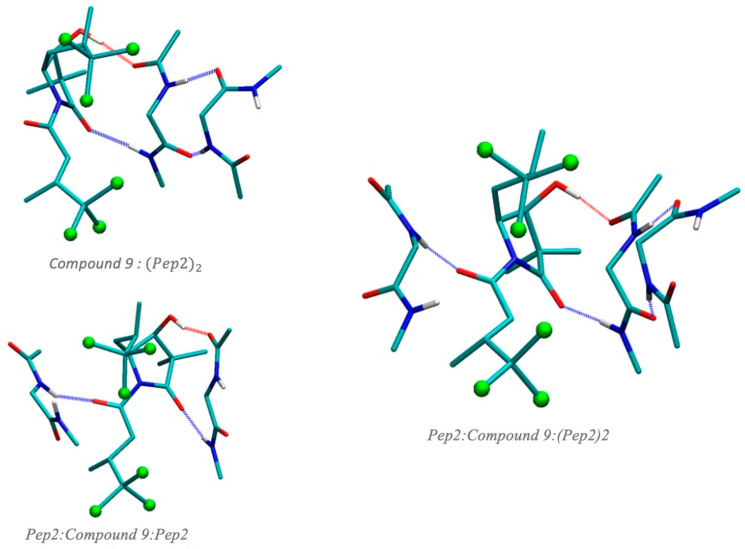
Optimized structures at B3LYP-B3DJ(PCM)/6-31+G(d,p) for the interaction of compound **9** with two glycine dipeptides (Pep2), namely compound **9** interacting with Pep2 bound in ß-sheet antiparallel conformation to another Pep2 (Compound **9**:${\left(Pep2\right)}_{2}$ structure), compound **9** intercalated between two Pep2 (Pep2:Compound **9**:Pep2), and a compound **9** hydrogen bound to two Pep2 dipeptides interacting in a ß-sheet antiparallel conformation, and a Pep2 dipeptide opposite to the ß-sheet (Pep2:Compound **9**:(Pep2)2). Hydrogens that do not participate in hydrogen bonds are omitted for clarity.

**Figure 8:**
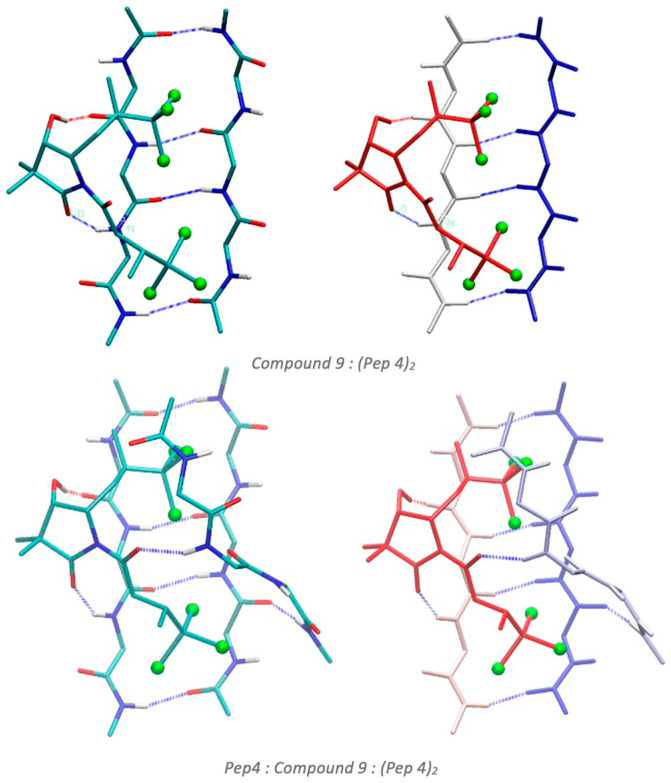
Optimized structures at the B3LYP-B3DJ(PCM)/6-31+G(d,p) for the interaction of compound **9** with four glycine peptides (Pep4), namely compound **9** interacting with two Pep4 bound in ß-sheet antiparallel conformation (Compound **9**: ${\left(Pep4\right)}_{2}$ structure), and a compound **9** hydrogen bound to two Pep4 that form a ß-sheet antiparallel structure and a Pep4 opposite to the ß-sheet (Pep4: Compound **9**:$\;{\left(Pep4\right)}_{2}$). Hydrogens that do not participate in hydrogen bonds are omitted for clarity.

To analyze the inherent propensity of compounds **9** and **10** to interact with the backbone of the peptides, we performed accurate quantum chemical calculations summarized in Table 3 and Figure 5, Figure 6, Figure 7 and Figure 8. Figure 5 depicts the interaction energies between key functional groups of peptides and compounds **9** and **10**. The interactions correspond to hydrogen bonds between different functional groups present in compound **9** or compound **10** and the peptide bonds. We observed many stabilizing hydrogen bond interactions between the peptide group and the carbonyl and hydroxyl functional groups present in compounds **9** and **10**. Thus, the hydrogen bond between a carbonyl group and the peptide (Pep:Carb) is −7.4 kcal/mol, whereas the hydroxyl group presents two possible modes of interaction with high stabilizing effects, namely, −5.4 kcal/mol for the hydrogen bond interaction with the H-N side of the peptide bond (Pep:Met), and −6.7 kcal/mol with the carbonyl side (Pep:Met_a structure). Noteworthy, the presence of the chlorine atoms also leads to significant stabilization by interaction with the peptide π-bond, −3.6 kcal/mol. We also provide as reference the interaction energy of two glycine dipeptides interacting through hydrogen bonds in a β-sheet conformation (Pep2_2_) with two hydrogen bonds accounting for −14.3 kcal/mol, whereas when we extend the model to cover tetrapeptides (Pep4_2_), with four hydrogen bonds between the two chains the interaction energy is −20.6 kcal/mol.

In Figure 6, we considered the interaction of compounds **9** and **10** with a glycine dipeptide (Pep2) using two models. In one model (C9m and C10m), we only considered the central part of the compounds without the aliphatic halogenated chains. We can see that both compounds C9m and C10m show the carbonyl and hydroxyl groups at an appropriate distance to interact optimally through hydrogen bonds with the Pep2 backbone peptide when adopting a β-sheet configuration, with interaction energies of −14.9 for C10m:Pep2, and −11.9 kcal/mol for C9m:Pep2. On the other hand, when the full model of compounds **9** and **10** are used, the interactions increase to −19.3 and −18.1 kcal/mol for compound **10**:Pep2 and compound **9**:pep2, respectively, indicating the important stabilizing effect of the halogen atoms. Noteworthy is that these interactions are of the same magnitude as the ones expected for hydrogen bonds in Pep2_2_, indicating the high ability of these compounds to interact with these types of β-sheet structures, as observed experimentally.

Next, we considered the possibility of compound **9** interacting with a β-sheet dipeptide (Pep2_2_). The optimized structures are depicted in Figure 7. First, we considered the addition of compound **9** to Pep2_2_ without altering the β-sheet structure (compound **9**:Pep2_2_ in Figure 7). Energetics of these processes can be seen in Table 3. We can observe the high capacity of compound **9** to interact with Pep2_2_, −18.7 kcal/mol. Moreover, if compound **9** is interacting with one of the peptides, compound **9**:Pep2 does not alter the capacity for the peptide to interact with the second dipeptide, with an interaction of −14.9 kcal/mol, which is slightly higher than the one shown in Figure 5 for Pep2_2_. Thus, our results suggest that compound **9** shows an ability to interact with β-sheet structures without altering the hydrogen bond network established by the peptide at which compound **9** is attached with other peptides opposite to the direction of compound **9** bonding.

On the other hand, we also analyzed the capacity of compound **9** to be intercalated between the two chains of Pep2_2_. The reaction energy for the intercalation, namely $Compound\;9+{\left(Pep2\right)}_{2}\to Pep2:Compound\;9:Pep2$ is also quite exothermic, −15.2 kcal/mol. A key aspect of the capacity of this insertion is the presence of a carbonyl group in compound **9** that interacts with the Pep2 peptide. In the case of compound **10**, this carbonyl group participates in an intramolecular hydrogen bond (Figure 6) with an HN group and, thus, is unavailable for interaction with a peptide.

Therefore, compound **9**, on the one hand, has the ability to interact with a Pep2_2_ β-sheet structure without altering its intramolecular hydrogen bonding pattern, and in addition, provides an anchoring carbonyl group to interact with other chains. To confirm this, we calculated a Pep2: Compound **9**:Pep2_2_ structure. Energetics shown in Table 3 confirm the stability of such structures, with compound **9** having a high capacity to interact with the peptides.

Finally, we extended our models to cover glycine tetrapeptides (Figure 8), optimizing two types of structures: (i) compound **9** interacting with two glycine tetrapeptides interacting in a β-sheet conformation (Compound **9**:Pep4_2_ structure), and (ii) compound **9** interacting with the Pep4_2_ β-sheet structure and another Pep4 tetrapeptide chain opposite to the β-sheet, hydrogen bonded to the carbonyl group of compound **9** (Pep4: Compound **9**:Pep4_2_ structure). Energetics are shown in Table 3. As observed for dipeptides, there is a high capacity of compound **9** to interact with these tetrapeptide β-sheet structures by hydrogen bonds and interactions between the halogen atoms and the peptide π-bonds. Therefore, we obtain very favorable interaction energies that unequivocally show the capacity of compound **9** to interact with these peptides acting as an aggregating agent.

**Figure 9:**
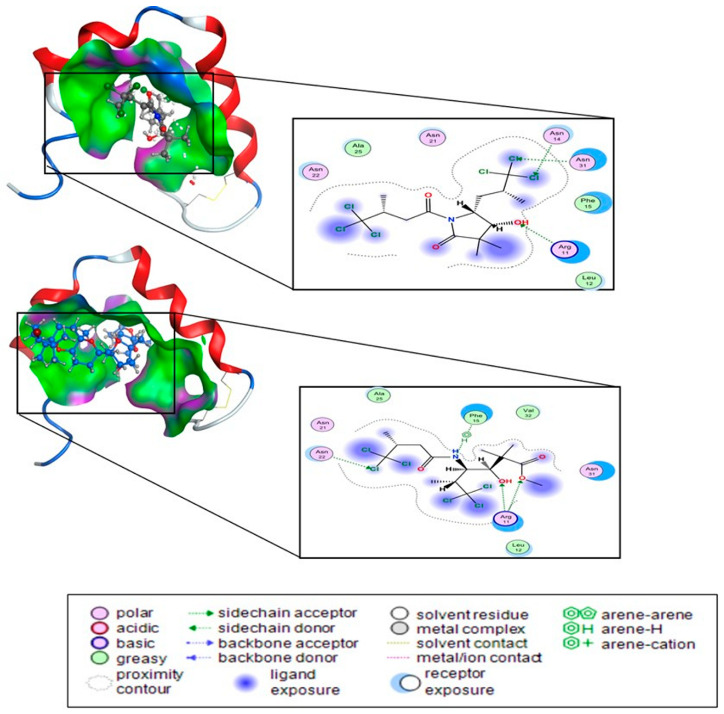
Protein-ligand interaction (2D and 3D) diagrams for compounds **9** and **10**. Purple color indicates hydrogen bond interactions, while green signifies hydrophobic interactions.

Molecular docking was used to investigate the role of other binding types on the aggregation kinetics of amylin and to understand their impact on structural behavior. The docking scores correlated with the experimental results, with compounds **9** and **10** exhibiting good scores of −5.91 and −6.156 kcal/mol, respectively. Figure 9 represents the ligand interaction in 2D and 3D of these compounds. The Cl atoms and OH group showed convincing interaction roles in both compounds. Compound **9** has strong hydrogen bonding with residues ARG 11, ASN 14, and ASN 31 (−1.6, −0.8, and −0.7 kcal/mol, receptively). Moreover, compound **10** accepts four hydrogen bonds from ARG 11, ASN22, and the 6-ring in PHE 15 residues from hIAPP. The energy for these bonds was −0.5 kcal/mol. Besides the hydrogen bonds, compounds **9** and **10** are surrounded by hydrophobic interactions presented in green color. All hydrogen bond interactions have a bond length of 2.76–3.52 Å.

## 5. Conclusions

This study investigated the effect of a chemically diverse panel of compounds isolated from marine macroorganisms of the Red Sea on hIAPP aggregation. β-Sheet formation, crucial for aggregation, relies on β-strand self-assembly through hydrogen bonding. Compounds **5** and **9** showed a significant increase in hIAPP fluorescence intensity, particularly during elongation, indicating their potential as aggregation promoters. Analysis of the structures of the investigated compounds revealed that hydrogen bond acceptors (e.g., oxygen or chlorine atoms) facilitate β-sheet formation, making the aggregation process easier by forming new hydrogen bonds. Compound **1** had minimal impact, while compounds **2**–**4**, **6**, and **8** actively promoted aggregation, with compound **6** initiating elongation after approximately 3.5 h. Molecular docking results were well correlated with experimental results and the interface scrutiny indicated that hydrophobic and hydrogen bond interactions synergistically play vital roles. These findings enhance our understanding of potential therapeutic strategies to tackle amyloid-related diseases based on natural products from Red Sea organisms. Further comprehensive studies are necessary to explore these compounds’ effects in various research and application areas.

## Figures and Tables

**Table 1:** Docking results for EGCG and compounds **1**–**10** against hIAPP (ID = 2L86).

Compound	S	rmsd_refine	E_conf	E_place	E_score1	E_refine	E_score2
EGCG	−6.50048	2.187413	−21.6101	−85.7432	−12.4433	−37.4309	−6.50048
**1**	−4.81217	1.235778	8.072261	−57.4571	−7.34267	−18.1921	−4.81217
**2**	−4.73856	1.180173	−51.9049	−58.0496	−7.55631	−20.8414	−4.73856
**3**	−5.3566	1.585066	167.585	−72.3839	−8.47299	−23.2986	−5.3566
**4**	−5.30706	2.052274	163.3808	−62.6056	−7.92134	−25.3408	−5.30706
**5**	−5.93741	2.702701	39.48293	−75.5513	−8.28225	−22.6939	−5.93741
**6**	−6.66936	2.166606	51.81665	−87.7867	−8.75448	−35.7981	−7.39696
**7**	−5.26524	0.594658	189.8514	−69.5336	−8.01973	−25.9222	−5.26524
**8**	−5.57006	1.773319	55.72262	−59.4908	−7.73278	−25.3146	−5.57006
**9**	−5.75957	1.795054	46.90462	−72.7427	−8.7276	−29.8373	−5.90957
**10**	−5.93587	1.286014	41.9398	−87.0925	−7.75724	−28.8094	−6.15587

**Table 2:** Effect of EGCG and compounds **1**–**10** on hIAPP nucleation and elongation phases with regards to ThT results.

Compound	Δ Nucleation	Δ Elongation	Chemical Formula
EGCG	++	− −	C_22_H_18_O_11_
**1**	=	=	C_15_H_24_O
**2**	+	+	C_15_H_20_O_3_
**3**	++	++	C_20_H_30_O_2_
**4**	++	++	C_20_H_28_O_3_
**5**	++	+++	C_30_H_52_O_4_
**6**	+++	++	C_32_H_55_BrO_8_
**7**	+	−	C_15_H_20_Br_2_O_3_
**8**	++	++	C_15_H_20_Br_2_O_2_
**9**	+++	++++	C_15_H_21_Cl_6_NO_3_
**10**	−	−	C_16_H_25_Cl_6_NO_4_

**Table 3:** Reaction energies in aqueous solution (ΔEaq) for the interaction of compound **9** with different glycine peptides are depicted in Figure 7 and Figure 8. All energies are calculated in kcal/mol.

Reaction	ΔE_aq_
$Formation\;of\;{Compound\;9:\left(Pep2\right)}_{2}$	
$Compound\;9+2Pep2\to {Compound\;9:\left(Pep2\right)}_{2}$	−32.9
$Compound\;9:Pep2+Pep2\to {Compound\;9:\left(Pep2\right)}_{2}$	−14.9
$Compound\;9+{\left(Pep2\right)}_{2}\to {Compound\;9:\left(Pep2\right)}_{2}$	−18.7
Formation of Pep2:Compound 9:Pep2	
Compound 9+2Pep2→Pep2:Compound 9:Pep2	−29.5
Pep2+Compound 9:Pep2→Pep2:Compound 9:Pep2	−11.4
$Compound\;9+{\left(Pep2\right)}_{2}\to Pep2:Compound\;9:Pep2$	−15.2
$Formation\;of\;Pep2:Compound\;9:{\left(Pep2\right)}_{2}$	
$Compound\;9+3Pep2\to Pep2:Compound\;9:{\left(Pep2\right)}_{2}$	−43.6
$Pep2+Compound\;9:{\left(Pep2\right)}_{2}\to Pep2:Compound\;9:{\left(Pep2\right)}_{2}$	−10.6
$Pep2:Compound\;9:Pep2+Pep2\to Pep2:Compound\;9:{\left(Pep2\right)}_{2}$	−14.1
${Formation\;of\;Compound\;9:\left(Pep4\right)}_{2}$	
$Compound\;9+2Pep4\to {Compound\;9:\left(Pep4\right)}_{2}$	−48.3
$Compound\;9:Pep4+Pep4\to {Compound\;9:\left(Pep4\right)}_{2}$	−26.4
$Compound\;9+{\left(Pep4\right)}_{2}\to {Compound\;9:\left(Pep4\right)}_{2}$	−27.7
$Formation\;of\;Pep4:Compound\;9:{\left(Pep4\right)}_{2}$	
$Compound\;9+3Pep4\to Pep4:Compound\;9:{\left(Pep4\right)}_{2}$	−66.4
$Pep4+Compound\;9:{\left(Pep4\right)}_{2}\to Pep4:Compound\;9:{\left(Pep4\right)}_{2}$	−18.2

## Data Availability

The original contributions presented in the study are included in the article. Further inquiries can be directed to the corresponding authors.
